# Evaluation of algorithms for Multi-Modality Whole Heart Segmentation: An open-access grand challenge

**DOI:** 10.1016/j.media.2019.101537

**Published:** 2019-12

**Authors:** Xiahai Zhuang, Lei Li, Christian Payer, Darko Štern, Martin Urschler, Mattias P. Heinrich, Julien Oster, Chunliang Wang, Örjan Smedby, Cheng Bian, Xin Yang, Pheng-Ann Heng, Aliasghar Mortazi, Ulas Bagci, Guanyu Yang, Chenchen Sun, Gaetan Galisot, Jean-Yves Ramel, Thierry Brouard, Qianqian Tong, Weixin Si, Xiangyun Liao, Guodong Zeng, Zenglin Shi, Guoyan Zheng, Chengjia Wang, Tom MacGillivray, David Newby, Kawal Rhode, Sebastien Ourselin, Raad Mohiaddin, Jennifer Keegan, David Firmin, Guang Yang

**Affiliations:** a School of Data Science, Fudan University, Shanghai, 200433, China; b Fudan-Xinzailing Joint Research Center for Big Data, Fudan University, Shanghai, 200433, China; c School of Biomedical Engineering, Shanghai Jiao Tong University, Shanghai, 200240, China; d Institute of Computer Graphics and Vision, Graz University of Technology, Graz, 8010, Austria; e Ludwig Boltzmann Institute for Clinical Forensic Imaging, Graz, 8010, Austria; f Institute of Medical Informatics, University of Lubeck, Lubeck, 23562, Germany; g Inserm, Université de Lorraine, IADI, U1254, Nancy, France; h Department of Biomedical Engineering and Health Systems, KTH Royal Institute of Technology, Stockholm SE-14152, Sweden; i School of Biomed. Eng., Health Science Centre, Shenzhen University, Shenzhen, 518060, China; j Dept. of Comp. Sci. and Eng., The Chinese University of Hong Kong, Hong Kong, China; k Center for Research in Computer Vision (CRCV), University of Central Florida, Orlando, 32816, U.S.; l School of Computer Science and Engineering, Southeast University, Nanjing, 210096, China; m LIFAT (EA6300), Université de Tours, 64 avenue Jean Portalis, Tours, 37200, France; n School of Computer Science, Wuhan University, Wuhan, 430072, China; o Guangdong Provincial Key Laboratory of Computer Vision and Virtual Reality Technology, SIAT, Shenzhen, China; p Shenzhen Institutes of Advanced Technology, Chinese Academy of Sciences, Shenzhen, 518055, China; q Institute for Surgical Technology & Biomechanics, University of Bern, Bern, 3014, Switzerland; r BHF Centre for Cardiovascular Science, University of Edinburgh, Edinburgh, U.K.; s Edinburgh Imaging Facility QMRI, University of Edinburgh, Edinburgh, U.K.; t School of Biomedical Engineering and Imaging Sciences, Kings College London, London, U.K.; u Cardiovascular Research Centre, Royal Brompton Hospital, London, SW3 6NP, U.K.; v National Heart and Lung Institute, Imperial College London, London, SW7 2AZ, London, U.K.

**Keywords:** Whole Heart Segmentation, Multi-modality, Benchmark, Challenge

## Abstract

Knowledge of whole heart anatomy is a prerequisite for many clinical applications. Whole heart segmentation (WHS), which delineates substructures of the heart, can be very valuable for modeling and analysis of the anatomy and functions of the heart. However, automating this segmentation can be challenging due to the large variation of the heart shape, and different image qualities of the clinical data. To achieve this goal, an initial set of training data is generally needed for constructing priors or for training. Furthermore, it is difficult to perform comparisons between different methods, largely due to differences in the datasets and evaluation metrics used. This manuscript presents the methodologies and evaluation results for the WHS algorithms selected from the submissions to the Multi-Modality Whole Heart Segmentation (MM-WHS) challenge, in conjunction with MICCAI 2017. The challenge provided 120 three-dimensional cardiac images covering the whole heart, including 60 CT and 60 MRI volumes, all acquired in clinical environments with manual delineation. Ten algorithms for CT data and eleven algorithms for MRI data, submitted from twelve groups, have been evaluated. The results showed that the performance of CT WHS was generally better than that of MRI WHS. The segmentation of the substructures for different categories of patients could present different levels of challenge due to the difference in imaging and variations of heart shapes. The deep learning (DL)-based methods demonstrated great potential, though several of them reported poor results in the blinded evaluation. Their performance could vary greatly across different network structures and training strategies. The conventional algorithms, mainly based on multi-atlas segmentation, demonstrated good performance, though the accuracy and computational efficiency could be limited. The challenge, including provision of the annotated training data and the blinded evaluation for submitted algorithms on the test data, continues as an ongoing benchmarking resource via its homepage (www.sdspeople.fudan.edu.cn/zhuangxiahai/0/mmwhs/).

## Highlights

• This work presents the methodologies and evaluation results for the WHS algorithms selected from the submissions to the Multi-Modality Whole Heart Segmentation (MM-WHS) challenge, in conjunction with MICCAI 2017.
• This work introduces the related information to the challenge, discusses the results from the conventional methods and deep learning-based algorithms, and provides insights to the future research.
• The challenge provides a fair and intuitive comparison framework for methods developed and being developed for WHS.
• The challenge provides the training datasets with manually delineated ground truths and evaluation for an ongoing development of MM-WHS algorithms.

## Graphical abstract

**Figure:**
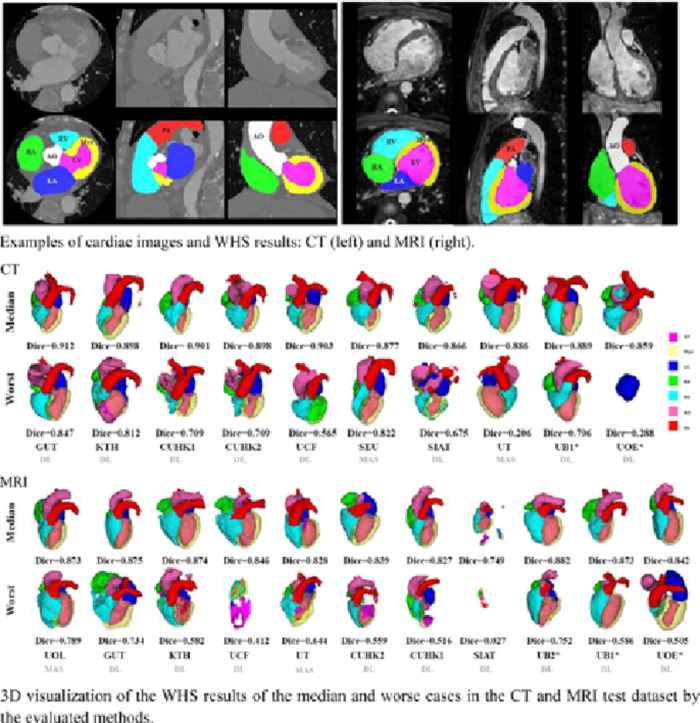

This manuscript presents the methodologies and evaluation results for the WHS algorithms selected from the submissions to the Multi-Modality Whole Heart Segmentation (MMWHS) challenge, in conjunction with MICCAI-STACOM 2017. The challenge provides 120 three-dimensional cardiac images covering the whole heart, including 60 CT and 60 MRI volumes, all acquired in clinical environments with manual delineation. Ten algorithms for CT data and eleven algorithms for MRI data, submitted from twelve groups, have been evaluated. The results show that many of the deep learning (DL) based methods achieved high accuracy, even though the number of training datasets were limited. Several of them also reported poor results in the blinded evaluation, probably due to over fitting in their training. The conventional algorithms, mainly based on multi-atlas segmentation, demonstrated robust and stable performance, even though the accuracy is not as good as the best DL method in CT segmentation. The challenge, including provision of the annotated training data and the blinded evaluation for submitted algorithms on the test data, continues as an ongoing benchmarking resource.

## 1 Introduction

According to the World Health Organization, cardiovascular diseases (CVDs) are the leading cause of death globally (Mendis et al., 2011). Medical imaging has revolutionized modern medicine and healthcare, and imaging and computing technologies have become increasingly important for the diagnosis and treatments of CVDs. Computed tomography (CT), magnetic resonance imaging (MRI), positron emission tomography (PET), single photon emission computed tomography (SPECT), and ultrasound (US) have been used extensively for physiologic understanding and diagnostic purposes in cardiology (Kang et al., 2012). Among these, CT and MRI are particularly used to provide clear anatomical information of the heart. Cardiac MRI has the advantages of being free from ionizing radiation, acquiring images with good contrast between soft tissues and with relatively high spatial resolution (Nikolaou et al., 2011). In contrast, cardiac CT, though involves ionizing radiation, is fast, low cost, and generally of high quality (Roberts et al., 2008).

**Fig. 1:**
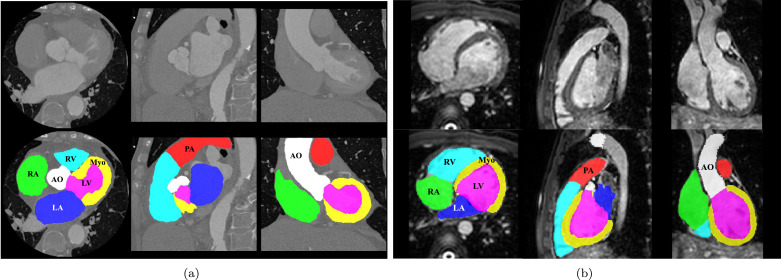
Examples of cardiac images and WHS results: (a) displays the three orthogonal views of a cardiac CT image and its corresponding WHS result, (b) shows example cardiac MRI data and the WHS result. LV: left ventricle; RV: right ventricle; LA: left atrium; RA: right atrium; Myo: myocardium of LV; AO: ascending aorta; PA: pulmonary artery.

To quantify the morphological and pathological changes, it is commonly a prerequisite to segment the important structures from the cardiac medical images. Whole heart segmentation (WHS) aims to extract each of the individual whole heart substructures, including the left ventricle (LV), right ventricle (RV), left atrium (LA), right atrium (RA), myocardium of LV (Myo), ascending aorta (AO) or the whole aorta, and the pulmonary artery (PA) (Zhuang, 2013), as Fig. 1 shows. The applications of WHS are numerous. The results can be used to directly compute the functional indices such as ejection fraction. Additionally, the geometrical information is useful in surgical guidance such as in radio-frequency ablation of the LA. However, the manual delineation of whole heart is labor-intensive and tedious, needing almost 8 hours for a single subject (Zhuang and Shen, 2016). Thus, automating the segmentation from multi-modality images, referred to as MM-WHS, is highly desired but still challenging, mainly due to the following reasons (Zhuang, 2013). First, the shape of the heart varies through the cardiac cycle as the heart contracts and relaxes. It also varies greatly from subject to subject, especially for those with pathological and physiological changes. Second, the appearance and image quality can be variable. For example, the enhancement patterns of the CT images can differ significantly for different scanners or acquisition sessions. Also, motion artifacts, poor contrast-to-noise ratio and signal-to-noise ratio, commonly presented in the clinical data, can significantly deteriorate the image quality and consequently challenge the task.

### 1.1 State-of-the-art for Whole Heart Segmentation

In the last ten years, a variety of WHS techniques have been proposed for cardiac CT and MRI data. Detailed reviews of previously published algorithms can be found in Kang et al. (2012), Zhuang (2013) and Peng et al. (2016). Kang et al. (2012) reviewed several modalities and corresponding segmentation algorithms for the diagnosis and treatments of CVDs. They summarized the roles and characteristics of different modalities of cardiac imaging and the parameter correlation between them. Furthermore, they categorized the WHS techniques into four, i.e., (1) boundary-driven techniques, (2) region-based techniques, (3) graph-cuts techniques, and (4) model fitting techniques. The advantages and disadvantages of each category were analyzed and summarized. Zhuang (2013) discussed the challenges and methodologies of the fully automatic WHS. Particularly, the work summarized two key techniques, i.e., the construction of prior models and the fitting procedure for segmentation propagation, for achieving this goal. Based on the types of prior models, the segmentation methods can be divided into two groups, namely the deformable model based methods and the atlas-based approaches. The fitting procedure can be decomposed into three stages, including localizing the whole heart, initializing the substructures, and refining the boundary delineation. Hence, this review paper by Zhuang (2013) mainly analyzes the algorithms based on the classification of prior models and fitting algorithms for the WHS from different modality images. Peng et al. (2016) reviewed both the methodologies of WHS and the structural and functional indices of the heart for clinical assessments. In their work, the WHS approaches were classified into three categories, i.e., image-driven techniques, model-driven techniques, and the direct estimation-based methods.

The three topic review papers mentioned above mainly cover publications before 2015. A collection of recent works not included by them are summarized in Table 1. Among these works, Zhuang et al. (2015) proposed an atlas ranking and selection scheme based on conditional entropy for the multi-atlas based WHS of CT. Zhou et al. (2017) developed a set of CT atlases labeled with 15 cardiac substructures. These atlases were then used for automatic WHS of CT via the multi-atlas segmentation (MAS) framework. Cai et al. (2017) developed a method with window width-level adjustment to pre-process CT data, which generates images with clear anatomical structures for WHS. They applied a Gaussian filter-based multi-resolution scheme to eliminate the discontinuity in the down-sampling decomposition for whole heart image registration. Zuluaga et al. (2013) developed a MAS scheme for both CT and MRI WHS. The proposed method ranked and selected optimal atlases based on locally normalized cross correlation. Pace et al. (2015) proposed a patch-based interactive algorithm to extract the heart based on a manual initialization from experts. The method employs active learning to identify the areas that require user interaction. Zhuang and Shen (2016) developed a multi-modality MAS framework for WHS of cardiac MRI, which used a set of atlases built from both CT and MRI. They proposed modality invariant metrics for computing the global image similarity and the local similarity. The global image similarity was used to rank and select atlases, from the multi-modality atlas pool, for segmenting a target image, and the local similarity metrics were proposed for the patch-based label fusion, where a multi-scale patch strategy was developed to obtain a promising performance.

**Table 1:** Summary of previous WHS methods for multi-modality images. PIS: patch-based interactive segmentation; FIMH: International Conference on Functional Imaging and Modeling of the Heart; MICCAI: International Conference on Medical Image Computing and Computer-assisted Intervention; MedPhys: Medical Physics; MedIA: Medical Image Analysis; RadiotherOncol: Radiotherapy and Oncology.

Reference	Data	Method	Runtime	Dice
Zuluaga et al. (2013), FIMH	8 CT, 23 MRI	MAS	60 min, 30 min	0.89 ± 0.04, 0.91 ± 0.03
Zhuang et al. (2015), MedPhys	30 CT	MAS	13.2 min	0.92 ± 0.02
Pace et al. (2015), MICCAI	20 MRI	PIS + Active learning	N/A	N/A
Zhuang and Shen (2016), MedIA	20 CT, 20 MRI	Multi-modality MAS	12.58 min	0.90 ± 0.03
Zhou et al. (2017), RadiotherOncol	31 CT	MAS	10 min	N/A
Cai et al. (2017), Neurocomputing	14 CT	Gaussian filter-based	N/A	N/A

In conclusion, WHS based on the MAS framework, referred to as MA-WHS, has been well researched in recent years. MAS segments an unknown target image by propagating and fusing the labels from multiple annotated atlases using image registration techniques. The performance relies on the registration algorithms for label propagation and the fusion strategy to combine the segmentation results from the multiple atlases. Both of these two key steps are generally computationally expensive.

Recently, deep learning (DL)-based methods have shown great promise in medical image analysis. They have achieved superior performance in various imaging modalities and different clinical applications (Roth, Lu, Seff, Cherry, Hoffman, Wang, Liu, Turkbey, Summers, 2014, Shen, Wu, Suk, 2017). For cardiac segmentation, Avendi et al. (2016) proposed a DL algorithm for LV segmentation. Ngo et al. (2017) trained multiple layers of a deep belief network to localize the LV, and to define the endocardial and epicardial borders, followed by the distance regularized level set. Recently, Tan et al. (2018) designed a fully automated convolutional neural network (CNN) architecture for pixel-wise labeling of both the LV and RV with impressive performance, and Mo et al. (2018) proposed a deep Poincare map-based method for LV segmentation. DL methods have the potential to provide faster and more accurate segmentation, compared to the conventional approaches, such as the deformable model based algorithms and MAS methods. However, little work has been reported to date using DL for WHS, probably due to the limitation of training data and complexity of the segmentation task.

### 1.2 Motivation and contribution

Due to the above mentioned challenges, we organized the competition of MM-WHS, providing 120 multi-modality whole heart images for developing new WHS algorithms, as well as validating existing ones. We also presented a fair evaluation and comparison framework for participants. In total, twelve groups who submitted their results and methods were selected, and they all agreed to contribute to this work, a benchmark for WHS of two modalities, i.e., CT and MRI. In this work, we introduce the related information, elaborate on the methodologies of these selective submissions, discuss the results and provide insights into future research.

The rest of this paper is organized as follows. Section 2 provides details of the materials and evaluation framework. Section 3 introduces the evaluated methods for benchmarking. Section 4 presents the results, followed by discussions in Section 5. We conclude this work in Section 6.

## 2 Materials and setup

### 2.1 Data acquisition

All the CT and MRI data have been anonymized in agreement with the local regional ethics committee before being released to the MM-WHS challenge, and they were acquired in real clinical environments. The cardiac CT/CTA data were obtained from two state-of-the-art 64-slice CT scanners (Philips Medical Systems, Netherlands) using a standard coronary CT angiography protocol at two sites in Shanghai, China. All the data cover the whole heart from the upper abdomen to the aortic arch. The in-plane resolution of the axial slices is 0.78 × 0.78 mm, and the average slice thickness is 1.60 mm. The cardiac MRI data were acquired from two hospitals in London, UK. One set of data was acquired from St. Thomas Hospital on a 1.5T Philips scanner (Philips Healthcare, Best, The Netherlands), and the other was from Royal Brompton Hospital on a Siemens Magnetom Avanto 1.5T scanner (Siemens Medical Systems, Erlangen, Germany). In both sites, a navigator-gated 3D balanced steady state free precession (b-SSFP) sequence was used for free-breathing whole heart imaging. The data were acquired at a resolution of around (1.6 ∼ 2) × (1.6 ∼ 2) × (2 ∼ 3.2) mm, and reconstructed to half of its acquisition resolution, i.e., about (0.8 ∼ 1) × (0.8 ∼ 1) × (1 ∼ 1.6) mm.

In total, we provided 120 multi-modality whole heart images from multiple sites, including 60 cardiac CT and 60 cardiac MRI. For each modality, we selected 20 images to form the training set, and the remaining 40 to form the test set. For the CT data, we used random sampling to divide the data into the two sets. This is because the CT data were acquired from the two sites of the same hospital, and the data were equally distributed to the two sites. For the MRI data, they were acquired from two different hospitals. One provided 19 images, and the other provided 41 images. We divided the data from each hospital into two subsets, one for training (about one third) and the other for test (about two thirds). We then combined them to form the training set of 20 cases and test set of 40 cases. The pathologies involved in the MRI data covered a wide range of cardiac diseases, including myocardium infarction, atrial fibrillation (AF), tricuspid regurgitation, aortic valve stenosis, Alagille syndrome, Williams syndrome, dilated cardiomyopathy, aortic coarctation, and Tetralogy of Fallot. For analyzing the WHS performance with respect to different pathologies, we divided them into three categories, i.e., congenital heart disease (CHD) cases, AF patients, and *Others*. The numbers of subjects of these three categories in the training set are respectively 7, 6 and 7, and the numbers in the test set are respectively 9, 13, 18. Please refer to Section 5.3 for details of discussion.

### 2.2 Definition and gold standard

The WHS in this work aims to delineate and extract the seven substructures of the heart (Zhuang, 2013). These are:

(1) The LV blood cavity, also referred to as the LV. The boundary between the LV and LA is defined by the plane of the mitral valve annulus, and the boundary between the LV and aorta is defined by the plane of the aortic valve annulus. The papillary muscles are included in the LV, according to the recommendation of cardiologists.
(2) The RV blood cavity, also referred to as the RV. The boundary between the RV and RA is defined by the plane of the tricuspid valve annulus, and the boundary between the RV and PA is defined by the plane of the pulmonary valve annulus.
(3) The LA blood cavity, also referred to as the LA. LA solely consists of the blood pool within the endocardium of the LA cavity, excluding the pulmonary veins (PVs) and left atrial appendage. The boundaries between the LA and PVs are determined by following each PV distally to the LA body and truncating at the point when there is no clear vein to follow (Tobon-Gomez et al., 2015).
(4) The RA blood cavity, also referred to as the RA. The boundaries between the RA and superior/ inferior vena cava are determined at the point when there is no clear vena cava to follow, similar to the definition of boundaries between the LA and PVs.
(5) The myocardium of the LV, referred to as the Myo. Myo has two surfaces, i.e., the epicardial surface (Epi) and the endocardial surface of the LV.
(6) The AO trunk from the aortic valve to the superior level of the atria, also referred to as the AO. In our training data, the provided manual segmentation generally covers the whole ascending aorta to include the aortic arch. This means the distal end of the segmented great vessel exceeds the cutting point of the definition. However, in the evaluation we only consider the major trunk by manually cutting off the part of aorta which exceeds the superior level of the atria. We do this to avoid biased evaluation due to the inconsistent definition of the distal end of a great vessel.
(7) The PA trunk from the pulmonary valve to the bifurcation point, also referred to as the PA. Similar to AO, for the training data we provide the manual segmentation which exceeds the distal end of the definition. However, for the test data we truncate the segmentation at the bifurcation point of the pulmonary artery before evaluating the accuracy of a result.

The four blood pool cavities, i.e., LV, RV, LA and RA, are also referred to as the four chambers.

Manual labeling was adopted for generating the gold standard segmentation. This was done slice-by-slice using the ITK-SNAP software (Yushkevich et al., 2006), either by clinicians or by students who majored in biomedical engineering or medical physics and were familiar with the whole heart anatomy. Each manual segmentation result was examined by a senior researcher specialized in cardiac imaging with experience of more than five years, and modifications were made where required. The sagittal and coronal views were visualized simultaneously to check the consistency and smoothness of the segmentation, although the manual delineation was mainly performed in the axial views. For each 3D image, it took approximately 6–10 h for the observer to complete the manual segmentation of the whole heart.

### 2.3 Evaluation metrics

We employed four widely used metrics to evaluate the accuracy of a segmentation result (Zhuang, 2013): the Dice score, Jaccard index, surface-to-surface distance (SD), and Hausdorff Distance (HD). For WHS evaluation, the generalized metrics were used, which are expected to be more objective (Crum, Camara, Hill, 2006, Zhuang, 2013).

For each modality, the data were split into two sets, i.e., the training set (20 CT and 20 MRI) and the test set (40 CT and 40 MRI). For the training data, both the images and the corresponding gold standard were released to the participants for building, training and cross-validating their models. For the test data, only the CT and MRI images were released. Once the participants developed their algorithms, they could submit their segmentation results on the test data to the challenge moderators for a final independent evaluation. To avoid parameter tuning via multiple submissions, the organizers only allowed a maximum of two evaluations of segmentation accuracies for one algorithm.

### 2.4 Participants

Twelve algorithms (teams) were selected for this benchmark work. Nine of them provided results for both CT and MRI data, one experimented only on the CT data and two worked solely on the MRI data.

All the 12 teams agreed to include their results in this paper. To simplify the description below, we used the team abbreviations referring to both the teams and their corresponding methods and results. The evaluated methods are elaborated on in Section 3, and the key contributions of the teams are summarized in Table 2. Note that the three methods, highlighted with an asterisk (*), were submitted after the deadline of the challenge. To be fair to the groups who submitted before the deadline, we excluded the late submissions from ranking and competing for the awards of the challenge. However, for this manuscript we include all the high quality submissions, to maximize the number of methods for benchmark and quality of the paper.

**Table 2:** Summary of submitted methods.

Teams	Tasks	Key elements in methods	Teams	Tasks	Key elements in methods
GUT	CT, MRI	Two-step CNN, combined with anatomical label configurations.	UOL	MRI	MAS and discrete registration, to adapt the large shape variations.
KTH	CT, MRI	Multi-view U-Nets combining hierarchical shape prior.	CUHK1	CT, MRI	3D fully connected network (FCN) with the gradient flow optimization and Dice loss function.
SEU	CT	Conventional MAS-based method.	CUHK2	CT, MRI	Hybrid loss guided FCN.
UCF	CT, MRI	Multi-object multi-planar CNN with an adaptive fusion method.	UT	CT, MRI	Local probabilistic atlases coupled with a topological graph.
SIAT	CT, MRI	3D U-Net network learn multi-modality features.	UB2⁎	MRI	Multi-scale fully convolutional Dense-Nets.
UB1⁎	CT, MRI	Dilated residual networks.	UOE⁎	CT, MRI	Two-stage concatenated U-Net.

⁎ Teams submitted results after the challenge deadline are indicated using Asterisk (*).

## 3 Evaluated methods

In this section, we elaborate on the twelve benchmarked algorithms. Table 2 provides the summary for reference.

### 3.1 Graz University of Technology (GUT)

Payer et al. (2017) propose a fully automatic whole heart segmentation, based on multi-label CNN and using volumetric kernels, which consists of two separate CNNs: one to localize the heart, referred to as localization CNN, and the other to segment the fine detail of the whole heart structure within a small region of interest (ROI), referred to as segmentation CNN. The localization CNN is designed to predict the approximate center of the bounding box around all heart substructures, based on the U-Net (Ronneberger et al., 2015) and heatmap regression (Payer et al., 2016). A fixed physical size ROI is then cropped around the predicted center, ensuring that it can enclose all interested substructures of the heart. Within the cropped ROI, the multi-label segmentation CNN predicts the label of each pixel. In this method, the segmentation CNN works on high-resolution ROI, while the localization CNN works on the low resolution images. This two-step CNN pipeline helps to mitigate the intensive memory and runtime generally required by the volumetric kernels equipped 3D CNNs.

### 3.2 University of Lubeck (UOL)

**Fig. 2:**
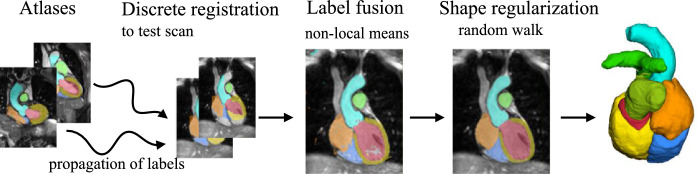
Multi-atlas registration and label fusion with regularization proposed by Heinrich and Oster (2017).

Heinrich and Oster (2017) propose a multi-atlas registration approach for WHS of MRI, as Fig. 2 shows. This method adopts a discrete registration, which can capture large shape variations across different scans (Heinrich et al., 2013b). Moreover, it can ensure the alignment of anatomical structures by using dense displacement sampling and graphical model-based optimization (Heinrich et al., 2013a). Due to the use of contrast-invariant features (Xu et al., 2016), the multi-atlas registration can implicitly deal with the challenging varying intensity distributions due to different acquisition protocols. Within this method, one can register all the training atlases to an unseen test image. The warped atlas label images are then combined by means of weighted label fusion. Finally, an edge-preserving smoothing of the generated probability maps is performed using the multi-label random walk algorithm, as implemented and parameterized in Heinrich and Blendowski (2016).

### 3.3 KTH Royal Institute of Technology (KTH)

Wang and Smedby (2017) propose an automatic WHS framework combining CNN with statistical shape priors. The additional shape information, also called shape context (Mahbod et al., 2018), is used to provide explicit 3D shape knowledge to the CNN. The method uses a random forest based landmark detection to detect the ROI. The statistical shape models are created using the segmentation masks of the 20 training CT images. The probability map is generated from three 2D U-Nets learned from the multi-view slices of the 3D training images. To estimate the shape of each subregion of heart, a hierarchical shape prior guided segmentation algorithm (Wang and Smedby, 2014) is then performed on the probability map. This shape information is represented using volumetric shape models, i.e., signed distance maps of the corresponding shapes. Finally, the estimated shape information is used as an extra channel, to train a new set of multi-view U-Nets for the final segmentation of the whole heart.

### 3.4 The Chinese University of Hong Kong, Method No. 1 (CUHK1)

Yang et al. (2017b) apply a general and fully automatic framework based on a 3D fully convolutional network (FCN). The framework is reinforced in the following aspects. First, an initialization is achieved by inheriting the knowledge from a 3D convolutional network trained on the large-scale Sports-1M video dataset (Tran et al., 2015). Then, the gradient flow is applied by shortening the back-propagation path and employing several auxiliary loss functions on the shallow layers of the network. This is to tackle the low efficiency and over-fitting issues when directly training the deep 3D FCNs, due to the gradient vanishing problem in shallow layers. Finally, the Dice similarity coefficient based loss function (Milletari et al., 2016) is included into a multi-class variant to balance the training for all classes.

### 3.5 University of Central Florida (UCF)

Mortazi et al. (2017a) propose a multi-object multi-planar CNN (MO-MP-CNN) method based on an encoder-decoder CNN. The multiple CNNs (Mortazi et al., 2017b) are trained from three different views, i.e., axial, sagittal, and coronal views, in 2D manners. An adaptive fusion method is then employed to combine the multiple outputs to refine the delineation. Furthermore, they apply a connected component analysis (CCA) on the final segmentation, to estimate the reliable (true positive) and unreliable (false positives) regions. Let *n* denote the number of classes in the images and *m* denote the number of components in each class, then the CCA could be performed as follows,

(1) $$\begin{array}{c} CCA\left(S\right)=\left\{S_{11},\cdots ,S_{nm}|\cup S_{ij}=o\right\}\&\\ \{S_{11},\cdots ,S_{nm}|\cap S_{ij}=\phi \}, \end{array}$$

where *S* indicates the segmentation result, *i* ∈ *m* and *j* ∈ *n*. The differences between the reliable and unreliable regions are used to guide the reliability of the segmentation process, namely the higher the difference, the more reliable the segmentation.

### 3.6 The Chinese University of Hong Kong, method no. 2 (CUHK2)

**Fig. 3:**
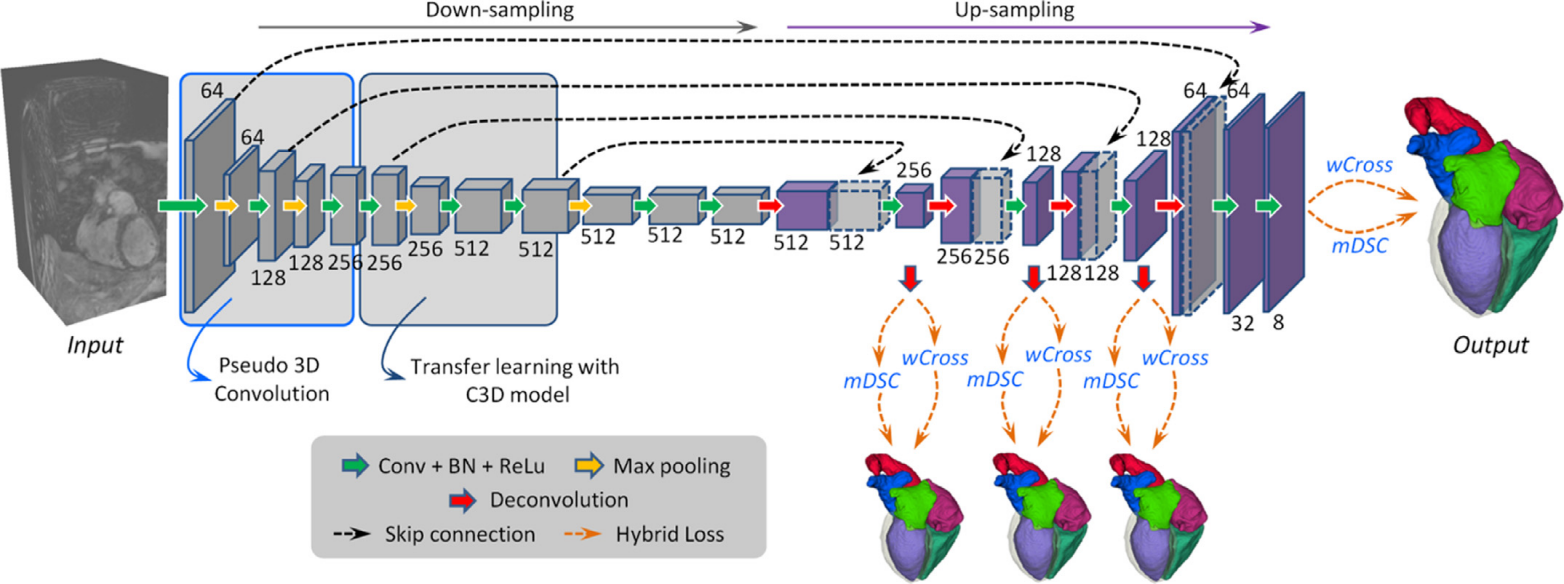
A schematic illustration of the method developed by Yang et al. (2017c). Digits represent the number of feature volumes in each layer. Volume with dotted line is for concatenation.

Yang et al. (2017c) propose to employ a 3D FCN for an end-to-end dense labeling, as Fig. 3 shows. The proposed network is coupled with several auxiliary loss functions in a deep supervision mechanism, to tackle the potential gradient vanishing problem and class imbalance in training. The network learns a spatial-temporal knowledge from a large-scale video dataset, and then transfer to initialize the shallow convolutional layers in the down-sampling path (Tran et al., 2015). For the class imbalance issue, a hybrid loss is proposed (Milletari et al., 2016), combining two complementary components: (1) volume-size weighted cross entropy loss (*wCross*) to preserve branch details such as the PA trunks. (2) multi-class Dice similarity coefficient loss (*mDSC*) to compact anatomy segmentation. Then, the proposed network can be well trained to simultaneously segment different heart substructures, and generate a segmentation in a dense but detail-preserved format.

### 3.7 Southeast University (SEU)

Yang et al. (2017a) develop a MAS-based method for WHS of CT images. The proposed method consists of the following major steps. Firstly, an ROI detection is performed on atlas images and label images, which are down-sampled and resized to crop and generate a heart mask. Then, an affine registration is used to globally align the target image with the atlas images, followed by a nonrigid registration to refine alignment of local details. In addition, an atlas ranking step is applied by using mutual information as the similarity criterion, and those atlases with low similarity are discarded. A non-rigid registration is further performed by minimizing the dissimilarity within the heart substructures using the adaptive stochastic gradient descent method. Finally, the propagated labels are fused with different weights according to the similarities between the deformed atlases and the target image.

### 3.8 University of Tours (UT)

Galisot et al. (2017) propose an incremental and interactive atlas-based WHS method, combining several local probabilistic atlases based on a topological graph. The training images are used to construct the probabilistic atlases, for each of the substructures of the heart. The graph is used to encode the priori knowledge to incrementally extract different ROIs. The priori knowledge about the shape and intensity distributions of substructures is stored as features to the nodes of the graph. The spatial relationships between these anatomical structures are also learned and stored as the edges of the graph. In the case of multi-modality data, multiple graphs are constructed, for example two graphs are built for the CT and MRI images, respectively. A pixelwise classification method combining hidden Markov random field is developed to integrate the probability map information. To correct the misclassifications, a post-correction is performed based on the Adaboost scheme.

### 3.9 Shenzhen Institutes of Advanced Technology (SIAT)

Tong et al. (2017) develop a deeply-supervised end-to-end 3D U-Net for fully automatic WHS. The training dataset are artificially augmented by considering each ROI of the heart substructure independently. To reduce false positives from the surrounding tissues, a 3D U-Net is first trained to coarsely detect and segment the whole heart structure. To take full advantage of multi-modality information so that features of different substructures could be better extracted, the cardiac CT and MRI data are fused. Both the size and the intensity range of the different modality images are normalized before training the 3D U-Net model. Finally, the detected ROI is refined to achieve the final WHS, which is performed by a pixel-wise classification fashion using the 3D U-Net.

### 3.10 University of Bern, method no. 1 (UB1*)

This method designs a voxelwise dilated residual network, referred as VoxDResNet, to segment the whole heart structures from 3D MRI images. It can be used to generate a semantic segmentation of an arbitrary-sized volume data after training. Conventional FCN methods integrate multi-scale contextual information by reducing the spatial resolution via successive pooling and sub-sampling layers, for semantic segmentation. By contrast, the proposed method achieves the same goal using dilated convolution kernels, without decreasing the spatial resolution of the network output. Additionally, residual learning is incorporated as pixel-wise dilated residual modules to alleviate the degrading problem, and the WHS accuracy can be further improved by avoiding gridding artifacts introduced by the dilation (Yu et al., 2017).

### 3.11 University of Bern, method no. 2 (UB2*)

This method includes a multi-scale pixel-wise fully convolutional Dense-Nets for 3D WHS of MRI images, which could directly map a whole volume of data to its volume-wise labels after training. The multi-scale context and multi-scale deep supervision strategies are adopted, to enhance feature learning. The deep neural network is an encoder (contracting path)-decoder (expansive path) architecture. The encoder is focused on feature learning, while the decoder is used to generate the segmentation results. Skip connection is employed to recover spatial context loss in the down-sampling path. To further boost feature learning in the contracting path, multi-scale contextual information is incorporated. Two down-scaled branch classifiers are inserted into the network to alleviate the potential gradient vanishing problem. Thus, more efficient gradients can be back-propagated from loss function to the shallow layers.

### 3.12 University of Edinburgh (UOE*)

Wang and Smedby (2017) develop a two-stage concatenated U-Net framework that simultaneously detects an ROI of the heart and classifies pixels into different substructures without losing the original resolution. The first U-Net uses a down-sampled 3D volume to produce a coarse prediction of the pixel labels, which is then re-sampled to the original resolution. The architecture of the second U-Net is inspired by the super-resolution CNN (SRCNN) (Dong et al., 2016) with skipping connections and recursive units (Kim et al., 2016). It inputs a two-channel 4D volume, consisting of the output of the first U-Net and the original data. In the test phase, a dynamic-tile layer is introduced between the two U-Nets to crop an ROI from both the input and output volume of the first U-Net. This layer is removed when performing an end-to-end training to simplify the implementation. Unlike the other U-Net based architecture, the proposed method can directly perform a prediction on the images with their original resolutions, thanks to the SRCNN-like network architecture.

## 4 Results

Tables 3 and 4 present the quantitative results of the evaluated algorithms on the CT and MRI datasets, respectively. The mean Dice scores of the evaluated methods for MM-WHS are respectively 0.872 ± 0.087 (CT) and 0.824 ± 0.102 (MRI), and the mean HDs are respectively 37.684 ± 17.026 mm (CT) and 39.209 ± 23.435 mm (MRI). In general, the evaluated algorithms obtain better WHS accuracies for CT than for MRI, using the four metrics. Section 5.2 provides a discussion of the difference between modalities.

**Table 3:** Results of the ten evaluated algorithms on CT dataset.

Teams	Dice	Jaccard	SD (mm)	HD (mm)	DL/MAS
GUT	**0.908 ± 0.086**	**0.832 ± 0.037**	**1.117 ± 0.250**	**25.242 ± 10.813**	DL
KTH	0.894 ± 0.030	0.810 ± 0.048	1.387 ± 0.516	31.146 ± 13.203	DL
CUHK1	0.890 ± 0.049	0.805 ± 0.074	1.432 ± 0.590	29.006 ± 15.804	DL
CUHK2	0.886 ± 0.047	0.798 ± 0.072	1.681 ± 0.593	41.974 ± 16.287	DL
UCF	0.879 ± 0.079	0.792 ± 0.106	1.538 ± 1.006	28.481 ± 11.434	DL
SEU	0.879 ± 0.023	0.784 ± 0.036	1.705 ± 0.399	34.129 ± 12.528	MAS
SIAT	0.849 ± 0.061	0.742 ± 0.086	1.925 ± 0.924	44.880 ± 16.084	DL
UT	0.838 ± 0.152	0.742 ± 0.161	4.812 ± 13.604	34.634 ± 12.351	MAS
UB1*	0.887 ± 0.030	0.798 ± 0.048	1.443 ± 0.302	55.426 ± 10.924	DL
UOE*	0.806 ± 0.159	0.697 ± 0.166	4.197 ± 7.780	51.922 ± 17.482	DL
Average	0.859 ± 0.108	0.763 ± 0.118	3.259 ± 9.748	34.382 ± 12.468	MAS
	0.875 ± 0.083	0.784 ± 0.010	1.840 ± 2.963	38.510 ± 17.890	DL
	0.872 ± 0.087	0.780 ± 0.102	2.124 ± 5.133	37.684 ± 17.026	ALL

**Table 4:** Results of the eleven evaluated algorithms on MRI dataset.

Teams	Dice	Jaccard	SD (mm)	HD (mm)	DL/MAS
UOL	0.870 ± 0.035	0.772 ± 0.054	1.700 ± 0.649	**28.535 ± 13.220**	MAS
GUT	0.863 ± 0.043	0.762 ± 0.064	1.890 ± 0.781	30.227 ± 14.046	DL
KTH	0.855 ± 0.069	0.753 ± 0.094	1.963 ± 1.012	30.201 ± 13.216	DL
UCF	0.818 ± 0.096	0.701 ± 0.118	3.040 ± 3.097	40.092 ± 21.119	DL
UT	0.817 ± 0.059	0.695 ± 0.081	2.420 ± 0.925	30.938 ± 12.190	MAS
CUHK2	0.810 ± 0.071	0.687 ± 0.091	2.385 ± 0.944	33.101 ± 13.804	DL
CUHK1	0.783 ± 0.097	0.653 ± 0.117	3.233 ± 1.783	44.837 ± 15.658	DL
SIAT	0.674 ± 0.182	0.532 ± 0.178	9.776 ± 6.366	92.889 ± 18.001	DL
UB2*	**0.874 ± 0.039**	**0.778 ± 0.060**	**1.631 ± 0.580**	28.995 ± 13.030	DL
UB1*	0.869 ± 0.058	0.773 ± 0.079	1.757 ± 0.814	30.018 ± 14.156	DL
UOE*	0.832 ± 0.081	0.720 ± 0.105	2.472 ± 1.892	41.465 ± 16.758	DL
Average	0.844 ± 0.047	0.734 ± 0.072	2.060 ± 0.876	29.737 ± 12.771	MAS
	0.820 ± 0.107	0.707 ± 0.127	3.127 ± 3.640	41.314 ± 24.711	DL
	0.824 ± 0.102	0.711 ± 0.125	2.933 ± 3.339	39.209 ± 23.435	ALL

For the CT data, the results are generally promising. The best Dice score (0.908 ± 0.086) and the best HD (25.242 ± 10.813 mm) were both achieved by GUT, which is a DL-based algorithm with anatomical label configurations. For the MRI data, the best Dice score (0.874 ± 0.039) was obtained by UB2*, which is a DL-based method and a delayed submission; and the best HD (28.535 ± 13.220 mm) was achieved by UOL, an MAS-based algorithm. Here, the average accuracy of MAS (two teams) was better than that of the DL-based segmentation (nine teams) in all evaluation metrics. However, the number of MAS-based approaches is limited, namely two, and the performance across different DL methods was variable, similar to the results from the CT experiment. For example, the top four DL methods by Dice scores, i.e., GUT, KTH, UB1* and UB2*, achieved comparable mean Dice scores to that of UOL (*p*=0.157, *p*=0.073, *p*=0.903 and *p*=0.448), but the other DL approaches generated much poorer results (*p* < 0.001). The discussion of different methodologies will be given in Section 5.4.

**Fig. 4:**
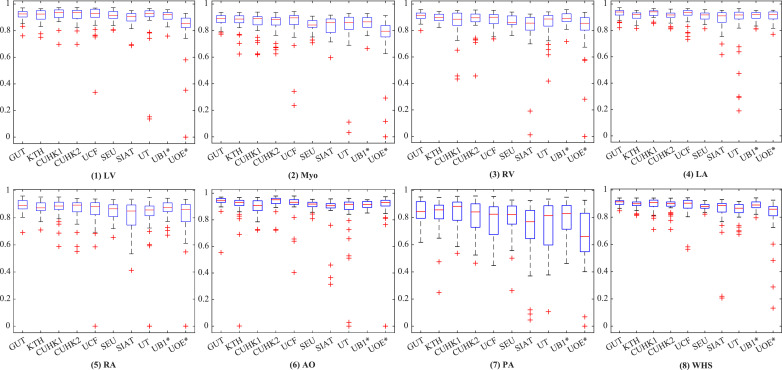
Boxplot of Dice scores of the whole heart segmentation on CT dataset by the ten methods.

**Fig. 5:**
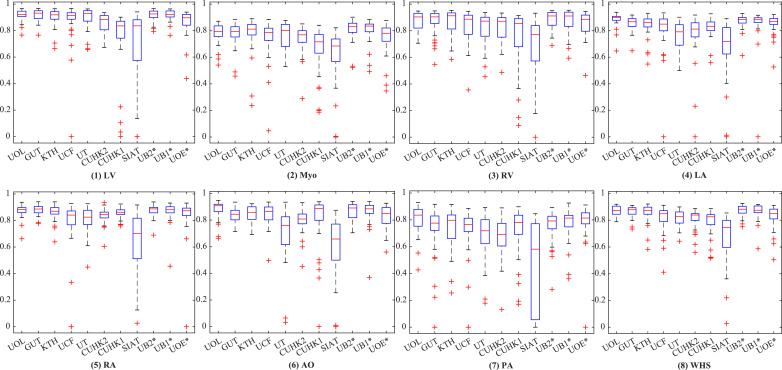
Boxplot of Dice scores of the whole heart segmentation on MRI dataset by the eleven methods.

Fig. 4 shows the boxplots of the evaluated algorithms on CT data. One can see that they achieved relatively accurate segmentation for all substructures of the heart, except for the PA whose variability in terms of shape and appearance is notably greater. For GUT, KTH, CUHK1, UB1*, and CUHK2, the delineation of PA is reasonably good with the mean Dice score larger than 0.80. Fig. 5 presents the boxplots on the MRI data. The five methods, i.e., UB2*, UOL, UB1*, GUT, and KTH, all demonstrate good Dice scores on the segmentation of four chambers and LV myocardium. Similar to the conclusion drawn from Tables 3 and 4, the segmentation on the CT images is generally better than that on the MRI data as indicated by the quantitative evaluation metrics.

**Fig. 6:**
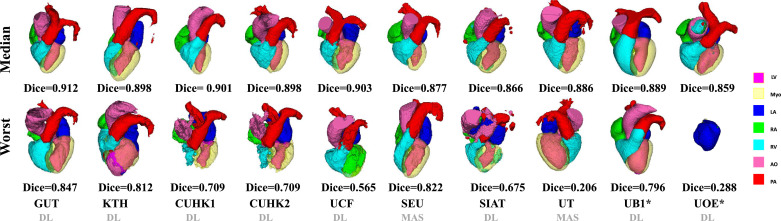
3D visualization of the WHS results of the median and worse cases in the CT test dataset by the ten evaluated methods. The color bar indicates the correspondence of substructures. Note that the colors of Myo and LV in 3D visualization do not look exactly the same as the keys in the color bar, due to the 50% transparency setting for Myo rendering and the addition effect from two colors (LV and 50% Myo) for LV rendering, respectively. (For interpretation of the references to colour in this figure legend, the reader is referred to the web version of this article.)

Fig. 6 shows the 3D visualization of the cases with the median and worst WHS Dice scores by the evaluated methods on the CT data. Most of the median cases look reasonably good, though some contain patchy noise; and the worst cases require significant improvements. Specifically, UOE* median case contains significant amount of misclassification in AO, and parts of the LV are labeled as LA in the UOE* and SIAT median cases. In the worst cases, the CUHK1 and CUHK2 results do not have a complete shape of the RV; KTH and SIAT contain a large amount of misclassification, particularly in myocardium; UCF mistakes the RA as LV; UOE* only segments the LA, and UT generates a result with wrong orientation.

**Fig. 7:**
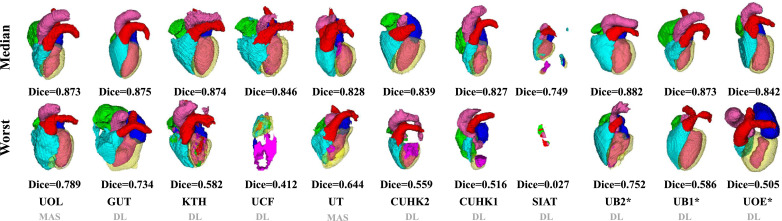
3D visualization of the WHS results of the median and worse cases in the MRI test dataset by the eleven evaluated methods.

Fig. 7 visualizes the median and worst results on MRI WHS. Compared with the CT results, even the median cases of MRI cases are poor. For example, the SIAT method could perform well on most of the CT cases, but failed to generate acceptable results for most of the MRI images, including the median cases presented in the figure. The worst cases of UOE*, CUHK2 and UB1 miss at least one substructure, and UCF and SIAT results do not contain any complete substructure of the whole heart. In conclusion, the CT segmentation results look better than the MRI results, which is consistent with the quantitative results. Also, one can see from Figs. 6 and 7 that the resulting shape from the two MAS-based methods looks more realistic, even though the segmentation could sometimes be very poor or even a failure, such as the worst MRI case by UOL and the worst CT case by UT.

The computational complexity of a DL method in the testing stage is related to the complexity of the network. In addition, DL methods can be implemented with the help of a GPU. Hence, the WHS of a case can be done within seconds or a minute on average. By contrast, the conventional approaches are commonly implemented with iterated optimization procedures, such as the atlas-to-target registration in the MAS, and thus could be computationally expensive. However, due to the difference of implementation and hardware an objective comparison between the evaluated methods can be difficult. For reference, we summarize the information regarding to the implementation details and their average run time in Table 5.

**Table 5:** Details on the average run time and computer systems used for the evaluated methods. T: average run time; Proc: average run time includes the pre- and post-processing of the images for the DL-based methods.

Teams	T (MRI)	T (CT)	Proc	GPU	CPU and RAM	Programming language
GUT	21 s	104 s	Y	GTX TITAN X; 12GB	Intel i7-4820K; 32GB	Python, C++
UOL	N/A	N/A	N/A	N/A	N/A	N/A
KTH	7 min	5 min	Y	GTX1080; 8GB	Intel Xeon E5 1620; 32GB	Python, C++
CUHK1	68.55 s	87.38 s	N	TITAN X (PASCAL); 12GB	Intel i5-6500; 16GB	Python + TensorFlow
SEU	N/A	20 min	N/A	N/A	Intel 7900X; 16G	Python + Elastix
CUHK2	66.03 s	89.79 s	N	TITAN X (PASCAL); 12GB	Intel i5-6500; 16GB	Python + TensorFlow
UCF	17 s	50 s	N	TITAN XP; 12GB	Intel Xeon E5-2630 v3; N/A	Python + TensorFlow
UT	14 min	21 min	N/A	N/A	Intel Core i7-4600; 16GB	C++, Cli
SIAT	7 s	11 s	N	GTX TITAN X; 12GB	Intel Core i5-7640X; 32GB	Python
UB2*	30 s	N/A	N	GTX 1080 Ti; 11GB	Intel(R) i7; 32GB	Python + TensorFlow
UB1*	28 s	23 s	N	GTX 1080 Ti; 11GB	Intel(R) i7; 32GB	Python + TensorFlow
UOE*	0.11 s	0.22 s	N	Telsa K80; 24GB	Intel Xeon E5-2686 v4; 64GB	Python + TensorFlow

## 5 Discussion

### 5.1 Overall performance of the evaluated algorithms

The segmentation accuracies reported for the four chambers are generally good, but the segmentation of the other substructures demonstrates more challenges. For example, one can see from Figs. 4 and 5 that in CT WHS the PA segmentation is much poorer compared to other substructures; in MRI WHS, the segmentation of myocardium, AO and PA appears to be more difficult. One reason could be that these regions have much larger variation in terms of shapes and image appearance across different scans. Particularly, the diverse pathologies can result in heterogeneous intensity of the myocardium and the blood, please refer to Section 5.3 for a detailed discussion.

Another reason could be the ambiguity in the manual delineations which are used as the ground truth for training of learning-based algorithms. This is likely to be greater for MR data than CT, as the image quality of whole heart MRI is generally lower (poorer contrast and signal-to-noise ratio). Table 6 shows the inter- and intra-observer variabilities in the manual delineation derived from a subset of MRI data. These are computed from the mean of 6 subjects (for inter-observer) and 4 subjects (for intra-observer), respectively. The inter-observer variabilities are comparable to the mean dice scores of the highest ranked methods (UOL and UB2*) in Table 4. Furthermore, the observer variation studies confirm that it can be more challenging to achieve consistent segmentation results on certain substructures, even for experienced observers. For example, in the variation studies the mean Dice scores of PA and Myo are much worse than those of the other substructures, particularly of LV and RV, which agrees with the different performance of the automatic methods in these substructures. Note that each of the gold standard segmentation used in this work was done by one rater, which is a limitation since the variability of manual segmentation between observers could be considerably large.

**Table 6:** The inter-observer (Inter-Ob) and intra-observer (Intra-Ob) variabilities of the MRI segmentation in Dice scores (%).

	LV	Myo	RV	LA
Inter-Ob	93.7 ± 1.33	81.1 ± 2.90	90.1 ± 1.96	83.7 ± 4.58
Intra-Ob	94.2 ± 0.84	83.9 ± 1.23	91.2 ± 2.59	86.8 ± 3.23
	RA	AO	PA	WHS
Inter-Ob	85.8 ± 3.10	87.6 ± 5.24	76.3 ± 14.34	87.8 ± 1.36
Intra-Ob	87.2 ± 2.48	91.1 ± 1.65	82.6 ± 3.77	89.5 ± 1.03

### 5.2 Discussion of different modalities: CT versus MRI

The MRI WHS is generally more challenging than the CT WHS, which is confirmed by the results presented in this work. The mean generalized Dice score of CT WHS is evidently better than that of MRI WHS averaged from the evaluated algorithms, namely 0.872 ± 0.087 (CT) versus 0.824 ± 0.102 (MRI), and *p*-value is 0.011 after the false discovery rate (FDR) correction (Benjamini et al., 2001). There is a significant difference between the mean HDs of the two modalities, namely 34.382 ± 12.468 mm (CT) versus 39.209 ± 23.435 mm (MRI) (*p* < 0.01, after FDR correction). One can further confirm this by comparing the results for these two tasks in Tables 3 and 4, as nine methods have been evaluated on both of the CT and MRI test data, and the same algorithms generally achieve better accuracies for CT data. Similar conclusion can also be drawn for the individual substructures as well as for the whole heart, when one compares the boxplots of segmentation Dice scores between Figs. 4 and 5.

### 5.3 Discussion of different pathologies

The pathologies of patients in this study cover a wide range of cardiac diseases. In particular, the MRI data include patients with CHD and AF, in whom the heart shape and size can vary considerably and in whom image quality can be more variable. We have therefore categorized the pathologies into three subgroups, i.e., CHD, AF and *Others*, and discuss the WHS performance for each.

The average WHS Dice scores of the evaluated methods on these three categories were respectively 0.817 ± 0.129 (CHD), 0.790 ± 0.103 (AF), 0.853 ± 0.072 (*Others*), as presented in Table 7. The *p*-values of the WHS Dice scores after FDR correction are as follows, *p*=0.001 between AF and CHD, *p*=0.005 between AF and *Others*, and *p*=0.017 between CHD and *Others*, indicating significant difference between these categories. One can see that the WHS result from the category of *Others* was evidently better than the other two with statistical significance.

**Table 7:** The performance of each substructure and WHS on different pathologies of the MRI in Dice scores (%).

	LV	Myo	RV	LA
AF	80.4 ± 17.9	71.8 ± 13.4	71.5 ± 15.8	84.4 ± 9.7
CHD	85.5 ± 16.6	69.6 ± 18.1	87.5 ± 11.3	78.2 ± 18.4
*Others*	91.2 ± 7.7	79.6 ± 8.2	88.6 ± 7.7	81.9 ± 1.14
	RA	AO	PA	WHS
AF	84.7 ± 10.1	76.5 ± 18.3	71.4 ± 20.7	79.0 ± 10.3
CHD	83.5 ± 11.3	80.9 ± 10.3	67.7 ± 23.4	81.7 ± 12.9
*Others*	81.2 ± 15.7	83.4 ± 13.4	73.4 ± 15.5	85.3 ± 7.2

For the CHD cohort, the evaluated methods tended to achieve less accurate results, especially in the substructures of LA, Myo and PA, probably due to large shape variations of the heart in these patients. For the AF patients, because of the irregular heart rhythm and shortness of breath, the image quality can be degraded, which could result in less accurate WHS results for the evaluated methods. Interestingly, we have found that the LA segmentation from AF patients was particularly more accurate (*p*=0.007, after FDR correction), and the ventricle segmentation, i.e., LV and RV, was much worse (*p*=0.025 and *p*=0.012, after FDR correction). This could be owing to the fact that the LA was larger for AF patients, and therefore could be easier to be recognized from the images by the algorithms. We therefore conclude that the segmentation of the substructures for different categories of patients can present different levels of challenges due to the difference in image quality and shape variations of the heart.

### 5.4 Discussion of different methodologies

As Tables 3 and 4 summarize, 9 out of the 11 benchmarked CT WHS methods and 8 out of the 10 MRI WHS algorithms are based on deep neural networks. Overall, the DL-based approaches have shown great potentials, particularly in CT WHS. However, several reported poor results based on their mean HDs as well as Dice scores, such as SIAT, UB1* and UOE* for CT WHS, and UCF, CUHK1 and SIAT for MRI WHS. The boxplots of Dice scores in Figs. 4 and 5 confirm that some of the DL methods have very large interquartile ranges and outliers. Figs. 6 and 7 visualize the 3D segmentation results of the median and the worst cases of each method. One can see that the resulting heart shapes of several cases are totally unrealistic, such as the worst CT case of UOE*, the median and worst MRI cases of SIAT, and the worst MRI cases of CUHK1 and UCF.

The performance of the DL methods could vary greatly across different network structures and training strategies, as summarized in Table 8. One can see that most of the DL-based approaches are 3D-based networks, except for KTH and UCF, which were based on multi-view 2D networks. The performance of 2D networks was comparable to that of 3D networks. For example, no significant difference was found between KTH and the two top performing 3D network-based methods, i.e., UB1* and GUT, in the MRI WHS Dice scores, as neither of the *p*-values was less than 0.1. This may be owing to the increased number of training data by using 2D networks, since one 3D image can be split into tens to hundreds of 2D slices. Additionally, the DL-based approaches are generally based on U-Net or FCN, except for UCF and UB2* which were based on encoder-decoder CNN. Two teams, i.e., CUHK1 and CUHK2, employed a pre-trained network to avoid overfitting. However, no significant difference was found between the different network architectures in terms of WHS Dice scores, and neither was there between the methods using pre-trained models and those which did not, as none of the *p*-values was less than 0.5. Only one team, i.e., KTH, embedded shape priors into the deep learning framework, which demonstrated good potential in improving the segmentation performance. Finally, SIAT was the only method to train the network using both the CT and MRI data, but the resulting network did not perform well. Hence, it is still an open question in terms of how to improve the generalization ability of a segmentation network by using multi-modality training data.

**Table 8:** Summary of the DL-based methods. The abbreviations are as follows, Dim: dimension; MS: multi-stage; E-D: encode-decode CNN; MM-train: trained on multi-modality datasets.

Teams	Dim	MS	Network	Prior	Pre-train	MM-train
GUT	3D	Y	U-Net	N	N	N
KTH	2D	Y	U-Net	Y	N	N
CUHK1	3D	N	FCN	N	Y	N
CUHK2	3D	N	FCN	N	Y	N
UCF	2D	N	E-D	N	N	N
SIAT	3D	Y	U-Net	N	N	Y
UB2*	3D	N	E-D	N	N	N
UB1*	3D	N	FCN	N	N	N
UOE*	3D	Y	U-Net	N	N	N

The conventional methods, mainly based on MAS in the evaluated methods, could generate stable results with more realistic shapes, though they were not necessarily competitive in terms of mean accuracies and computation efficiency. Particularly, in MRI WHS the MAS-based methods achieved no worse mean accuracies compared to the DL-based approaches, though only two MAS methods were submitted for evaluation. Finally, the advantages and potential limitations of all the evaluated methods are summarized in Table 9.

**Table 9:** Summary of the advantages and limitations of the twelve evaluated methods.

Method	Strengths	Limitations
GUT	- Combining localization and segmentation CNNs to reduce the requirements of memory and computation time. - Good segmentation performance for both CT and MRI.	- Based on an automatically localized landmark in the center of the heart, the cropping of a fixed physical size ROI is required for segmentation.
UOL	- The discrete registration can capture large shape variations across scans. - The regularization is used to obtain smooth surfaces that are important for mesh generation and motion or electrophysiological modelling.	- Only tested on the MRI data. - The automatic cropping of ROI sometimes do not cover the whole heart.
KTH	- Combining shape context information with orthogonal U-Nets for more consistent segmentation in 3-D views. - Good segmentation performance, particularly for CT.	- Potential of overfitting because the U-Nets rely much on the shape context channels. - Weighting factors of the shape context generation are determined empirically.
CUHK1	- Pre-trained 3-D Network provides good initialization and reduces overfitting. - Auxiliary loss functions are used to promote gradient flow and ease the training procedure. - Tackling the class-imbalance problem using a multi-class Dice based metric.	- The introduced hyperparameters need determining empirically. - Relatively poor performance in MRI WHS.
UCF	- Multi-planar information reinforce the segmentation along the three orthogonal planes. - Multiple 3-D CNNs require less memory compared to a 3-D CNN.	- The softmax function in the last layer could cause information loss due to class normalization.
CUHK2	- Coupling the 3-D FCN with transfer learning and deep supervision mechanism to tackle potential training difficulties caused by overfitting and vanishing gradient. - Enhance local contrast and reduce the image inhomogeneity.	- Relatively poor performance in MRI WHS.
SEU	- Three-step multi-atlas image registration method is lightweight for computing resources. - The method can be easily deployed.	- Only tested on the CT data.
UT	- The proposed incremental segmentation method is based on local atlases and allows users to perform partial and incremental segmentation.	- The registration of MRI atlas can be inaccurate, and the evaluated segmentation accuracy is low.
SIAT	- Combining a 3-D U-Net with a ROI detection to alleviate the impact of surrounding tissues and reduce the computational complexity. - Fusing MRI and CT images to increase the training samples and take full advantage of multimodality information so that features of different substructures can be better extracted.	- Poor segmentation performance, particularly for MRI data.
UB1*	- The focal loss and Dice loss are well encapsulated into a complementary learning objective to segment both hard and easy classes.	- Late submission of the WHS results.
UB2*	- Multi-scale context and multi-scale deep supervision are employed to enhance feature learning and to alleviate the potential gradient vanishing problem during training. - Reliable performance on the tested MR data.	- Late submission of the WHS results. - Only tested on the MRI data.
UOE*	- The proposed two-stage U-Net framework can directly segment the images with their original resolution.	- Late submission of the WHS results. - Poor performance, particularly for CT data.

### 5.5 Comparisons with the literature

Table 1 summarizes the WHS results from recent literature. Previous works were mainly based on conventional segmentation algorithms, such as MAS which achieved the most competitive performance before the introduction of DL-based methodologies. The best mean Dice score of WHS was around 0.90 for CT images from the literature, though it is important to notice that objective inter-work comparisons can be difficult, due to the difference in the evaluation metrics, implementations and study group pathologies. This is comparable to the results from the best performing methods in this challenge. For MRI data, both Zuluaga et al. (2013) and Zhuang and Shen (2016) reported a mean WHS Dice score of around 0.90, which is evidently better than the blinded evaluation results from this challenge. This could be attributed to the usage of the multi-modality atlases in the previous works, which improved the WHS by having more prior knowledge. By contrast, in this study only SIAT used multi-modality images to train their neural network, and the result was not promising. Hence, how to effectively train a neural network with multi-modality images remains an open question.

Table 10 summarizes the recent public datasets for cardiac segmentation, which mainly focus on specific substructures of the heart. Radau et al. (2009), Suinesiaputra et al. (2011), Petitjean et al. (2015) and Bernard et al. (2018) organized the challenges for segmenting the left, right or both ventricles. Moghari et al. (2016) organized a challenge for the segmentation of blood pool and myocardium from 3D MRI data. This work was aimed to offer pre-procedural planning of children with complex CHD. Karim et al. (2013), Tobon-Gomez et al. (2015), Karim et al. (2018) and Zhao and Xiong (2018) provided data for benchmarking algorithms of LA, LA wall, or LA scar segmentation for patients suffering from AF. Zhuang et al. (2019) organized a challenge for benchmarking the segmentation of ventricles and myocardium from multi-sequence cardiac MRI. Transfer learning or domain adaptation was particularly emphasized to achieve the segmentation of LGE MRI with the knowledge from other MRI sequences.

**Table 10:** Summary of the previous challenges related to cardiac segmentation from MICCAI society.

Organizers/refernece	Year	Data	Target	Pathology
Radau et al. (2009)	2009	45 cine MRI	LV	hypertrophy, infarction
Suinesiaputra et al. (2011)	2011	200 cine MRI	LV	myocardial infarction
Karim et al. (2013)	2013	60 MRI	LA scar	atrial fibrillation
Petitjean et al. (2015)	2012	48 cine MRI	RV	congenital heart disease
Tobon-Gomez et al. (2015)	2013	30 CT + 30 MRI	LA	atrial fibrillation
Karim et al. (2018)	2016	10 CT + 10 MRI	LA wall	atrial fibrillation
Moghari et al. (2016)	2016	20 MRI	Blood pool, Myo	congenital heart disease
Bernard et al. (2018)	2017	150 cine MRI	Ventricles	infarction, dilated/ hypertrophic
				cardiomyopathy, abnormal RV
Zhao and Xiong (2018)	2018	150 LGE-MRI	LA	atrial fibrillation
Zhuang et al. (2019)	2019	45 multi-modal MRI	Ventricles	cardiomyopathy

### 5.6 Progress and challenges

The MM-WHS challenge provides an open-access dataset and ongoing evaluation framework for researchers, to develop and compare their algorithms. Both the conventional methods and the new DL-based algorithms have made great progress, as shown in this paper. It is worth mentioning that the best performing DL methods have demonstrated great potential of generating accurate and reliable WHS results, such as GUT, UB1* and UB2*, even though they had limited training data (20 CT and 20 MRI). Despite this, there are limitations need to be overcome, particularly from the methodological point of view.

WHS of MRI is more challenging than that of CT. In general, the image quality of MRI data is poorer than that of CT data, in terms of contrast-to-noise ratio, signal-to-noise ratio and spatial resolution. In some patients, there can be blurring and/or ghosting artifacts due to the poorly corrected respiratory motion. In addition, the MRI datasets in this study included the particularly challenging cases from patients with CHD and AF. The former had large and challenging variations in cardiac anatomy, and the latter tented to have degraded image quality due to the irregular heart rhythm and shortness of breath of the patients. Enlarging the size of training data is commonly pursued to improve the learning-based segmentation algorithms. However, availability of whole heart training images can be difficult as well. One potential solution is to use artificial training data, such as by means of data augmentation or image synthesis using generative adversarial networks (Goodfellow et al., 2014). Furthermore, shape constraints can be incorporated into the training and prediction framework, which is particularly useful for the DL-based methods to avoid generating results of unrealistic shapes.

## 6 Conclusion

Knowledge of the detailed anatomy of the heart structure is clinically important as it is closely related to cardiac function and patient symptoms. Manual WHS is labor-intensive and also suffers from poor reproducibility. A fully automated multi-modality WHS is therefore highly in demand. However, achieving this goal is still challenging, mainly due to the variable quality of whole heart images, complex structure of the heart and large variation of the shape. This manuscript describes the MM-WHS challenge which provides 120 clinical CT/ MRI images, elaborates on the methodologies of twelve evaluated methods, and analyzes their results.

The challenge provides the same training data and test dataset for all the submitted methods. Note that these data are also open to researchers in future. The evaluation has been performed by the organizers, blind to the participants for a fair comparison. The results show that WHS of CT has been more successful than that of MRI from the twelve submissions. For segmentation of the substructures, the four chambers are generally easy to segment. By contrast, the great vessels, including aorta and pulmonary artery, still need more efforts to achieve good results. The performance of the DL-based methods submitted to this challenge was variable, with the best performing methods achieving high accuracy while the lowest performing methods were poor. The conventional atlas-based approaches generally performed well, though only 2 of the 11 MRI WHS methods and 2 of the 10 CT WHS algorithms submitted were none-DL-based. The hybrid methods, combining deep learning with prior information from either the multi-modality atlases or shape information of the heart substructures, should have good potential and be worthy of future exploration.

## Authors contributions

XZ initialized the challenge event, provided the 60 CT images, 41 MRI images (with KR and SO) of the 60 MRI images, and the manual segmentations of all the 120 images. GY, RM, JK, and DF provided the other 19 MRI images. XZ, GY and LL organized the challenge event, and LL evaluated all the submitted segmentation results. CP, DS, MU, MPH, JO, CW, OS, CB, XY, PAH, AM, UB, JB, GYu, CS, GG, JYR, TB, QT, WS, and XL were participants of the MM-WHS challenge and contributed equally. GZ, ZS, CW, TM and DN submitted their results after the deadline of the challenge. All the participants provided their results for evaluation and the description of their algorithms. All the authors have read and approved the publication of this work.

## Declaration of Competing Interest

We wish to confirm that there are no known conflicts of interest associated with this publication and there has been no signification financial support for this work that could have influenced its outcome. We confirm that the manuscript has been read and approved by all named authors and that there are no other of authors listed in the manuscript has been approved by all of us. We confirm that we have given due consideration to the protection of intellectual property associated with this work and that there are no impediments to publication, including the timing of publication, with respect to intellectual property. In so doing we confirm that we have followed the regulations of our institutions concerning intellectual property.
